# Artificial Intelligence in Alzheimer’s Disease Diagnosis and Prognosis Using PET-MRI: A Narrative Review of High-Impact Literature Post-Tauvid Approval

**DOI:** 10.3390/jcm14165913

**Published:** 2025-08-21

**Authors:** Rafail C. Christodoulou, Amanda Woodward, Rafael Pitsillos, Reina Ibrahim, Michalis F. Georgiou

**Affiliations:** 1 Department of Radiology, Stanford University School of Medicine, Stanford, CA 94305, USA; awoodward@stanford.edu; 2 Department of Neurophysiology, The Cyprus Institute of Neurology and Genetics, 2371 Nicosia, Cyprus; rafaelp@cing.ac.cy; 3 Faculty of Medicine, University of Balamand, Balamand 2807, Lebanon; reina.h.ibrahim@std.balamand.edu.lb; 4 Department of Radiology, Division of Nuclear Medicine, University of Miami, Miami, FL 33136, USA; mgeorgiou@med.miami.edu

**Keywords:** Alzheimer’s disease, artificial intelligence, MRI, PET, deep learning

## Abstract

**Background:** Artificial intelligence (AI) is reshaping neuroimaging workflows for Alzheimer’s disease (AD) diagnosis, particularly through PET and MRI analysis advances. Since the FDA approval of Tauvid, a PET tracer targeting tau pathology, there has been a notable increase in studies applying AI to neuroimaging data. This narrative review synthesizes recent, high-impact literature to highlight clinically relevant AI applications in AD imaging. **Methods:** This review examined peer-reviewed studies published between January 2020 and January 2025, focusing on the use of AI, including machine learning, deep learning, and hybrid models for diagnostic and prognostic tasks in AD using PET and/or MRI. Studies were identified through targeted PubMed, Scopus, and Embase searches, emphasizing methodological diversity and clinical relevance. **Results:** A total of 109 studies were categorized into five thematic areas: Image preprocessing and segmentation, diagnostic classification, prognosis and disease staging, multimodal data fusion, and emerging innovations. Deep learning models such as convolutional neural networks (CNNs), generative adversarial networks (GANs), and transformer-based architectures were widely employed by the research community in the field of AD. At the same time, several models reported strong diagnostic performance, but methodological challenges such as reproducibility, small sample sizes, and lack of external validation limit clinical translation. Trends in explainable AI, synthetic imaging, and integration of clinical biomarkers are also discussed. **Conclusions:** AI is rapidly advancing the field of AD imaging, offering tools for enhanced segmentation, staging, and early diagnosis. Multimodal approaches and biomarker-guided models show particular promise. However, future research must focus on reproducibility, interpretability, and standardized validation to bridge the gap between research and clinical practice.

## 1. Introduction

AD is considered the most prevalent neurodegenerative cause of dementia, with more than 60% of patients in a dementia outpatient clinic being diagnosed with AD [1]. The constant increase in elderly population due to the advances of medicine, combined with the rising incidence of Alzheimer’s disease in individuals over 65, makes AD a significant challenge for the healthcare community [2]. By the age of 85, the annual incidence of the disease rises to 7.6%, compared to the 0.4% in people between 65 and 74 years old [3]. Alzheimer’s Disease International reported in 2018 a global prevalence of 50 million people, with this number expected to triple by 2050 [1]. Despite the high incidence rate of AD, no effective treatment currently exists, and clinicians instead focus on improving patients’ quality of life and slowing the progression rate of dementia.

The pathophysiology underlying AD provides insights into the complexity of the brain networks that govern the higher cognitive functions. The first studies on the pathology of the disease during the early 1900s by Alois Alzheimer indicated the two pathological hallmarks of Alzheimer’s disease: extracellular amyloid-beta (Aβ) senile plaques, and intracellular neurofibrillary tangles (NFTs) [4]. The abnormal, insoluble form of Aβ protein is the product of the amyloid precursor protein (APP) cleavage by beta and gamma secretases. This leads to the highly amyloidogenic form of Aβ, which assembles into oligomers and accumulates extracellularly as plaques. The aggregations cause synaptic loss and eventually loss of neurons [5]. It is now widely accepted that the amyloid plaque deposition begins in the cerebrum years before the onset of cognitive decline and initiates the disease pathogenesis. Neurofibrillary tangles are formed due to tau protein hyperphosphorylation, a product of the MAPT gene, which contributes to neuronal degeneration by disrupting essential cellular processes [6]. Braak and Braak in 1991 proposed a neuropathological staging of AD that is consistent with the symptomatology of Alzheimer’s dementia [7]. Episodic memory impairment is most commonly the leading aspect of dementia, reflecting the damage occurring in the hippocampus. Executive dysfunction, attention deficits, and apathy develop as the disease progresses, indicating a more widespread cortical pathology [8].

Neuroimaging leverages these widespread pathological hallmarks of AD to enhance the diagnostic process and even predict patterns that appear in the late stages of the disease. Magnetic resonance imaging (MRI) and positron emission tomography (PET) are the most significant neuroimaging tools for AD diagnosis. MRI has historically been a powerful tool for detecting massive lesions and cerebrovascular events that contribute to cognitive decline, thereby ruling out neurodegeneration [9]. Recently, MRI has evolved into a precise and sensitive method for detecting atrophy patterns and volume loss caused by tissue degeneration [10]. PET scan, on the contrary, utilizes the metabolic dysfunction occurring in the diseased brain to detect the hypometabolism of the tissue and identify pathogenic accumulations in cells, Aβ, and tau [11]. With the FDA approval of Tauvid in 2020, a tau PET tracer, a groundbreaking advancement in Alzheimer’s diagnosis has been achieved. Tau tracers offer a novel diagnostic instrument for the clinical setting and catalyze a new wave of studies focusing on tau-specific detection [12].

The emerging high volume of imaging data generated by advanced and precise imaging techniques have presented significant challenges to the scientific community. The developing field of artificial intelligence (AI) illustrates an advancement of computing technology that “imitates” human intelligence by training a program to function humanely. Concurrently, artificial intelligence has emerged as a powerful tool for enhancing image interpretation, disease classification, and longitudinal prediction. In the Alzheimer’s research field, machine learning (ML) has been employed to detect MRI patterns by providing the system with data that is labeled (supervised learning) or unlabeled (unsupervised learning) and eventually predict unknown presented information, determining if the pattern exists or not [13]. Deep learning (DL), in contrast to ML, identifies the hidden patterns in complex imaging data using a hierarchy of neuronal networks, amplifying the relevant features and excluding the irrelevant information [14]. The fundamental distinction between the two AI subfields is that DL processes raw data without requiring human preprocessing, directly applying learned patterns to unknown inputs [15]. A multilayered network of nodes mimicking the human brain composes the artificial neuronal network (ANN). ANN uses ML and DL to analyze the input, process the data, and extract information on the classification of imaging and the prediction/prognosis of the disease [16].

As numerous novel technologies emerge in the groundbreaking field of AI applied in AD imaging, there is a growing need for guidance on their use and application to ensure a standardized implementation and optimized diagnostic accuracy. This review aims to narratively synthesize the highest-impact contributions in this area, offering clinicians and researchers a focused overview of AI-driven innovations in AD diagnosis using PET/MRI. Several novel technologies introduced in the last few years are highlighted in our study, with particular emphasis on their performance metrics and their implications for both research purposes and clinical applications. Although various reviews attempt to summarize the role of AI tools applied in neuroimaging or to provide insightful information regarding the use of AI in Alzheimer’s disease diagnosis [15,17], our review is among the first to comprehensively consolidate and critically assess high-impact AI applications in both PET and MRI for AD, thus offering an up-to-date and practice-oriented perspective that is currently lacking in the literature.

## 2. Materials and Methods

This narrative review highlights significant advancements in applying AI to diagnose and predict AD using PET, MRI, or a combination of both. The review focuses on studies published after the FDA approval of Tauvid, a PET tracer targeting tau pathology, which marked a pivotal milestone in AD neuroimaging.

A clinical research librarian (A.W.) conducted a comprehensive literature search using PubMed, Embase, and Scopus to identify peer-reviewed studies published between January 2020 and January 2025. The core search strategy focused on four key concepts: Alzheimer’s Disease, PET, MRI, and artificial intelligence, incorporating subterms such as “machine learning”, “deep learning”, “neural networks”, and related variants. Full database-specific search strategies are provided in Appendix A.1, Appendix A.2 and Appendix A.3.

To ensure a thorough and inclusive review, we also conducted manual citation screening. During this supplementary process, we applied additional keywords such as “radiomics”, “transformer”, “autoencoder”, “tau PET”, “fusion imaging”, “explainable AI”, and “multimodal deep learning” to identify high-impact studies not retrieved by the structured database queries.

We included only peer-reviewed original research articles and review papers that specifically focused on Alzheimer’s Disease, used AI techniques (machine learning, deep learning, or radiomics) that analyzed PET, MRI, or hybrid PET/MRI data. Studies were limited to those involving human participants and published in English. We excluded case reports, conference abstracts, editorials, non-peer-reviewed literature, studies on non-AD dementias, and those that used only traditional statistical methods without AI integration.

After deduplication and full-text screening, 109 studies met the inclusion criteria and were included in the final review. These studies were grouped into five thematic areas: (1) image preprocessing and segmentation, (2) diagnosis and classification, (3) prediction and prognosis, (4) multimodal data fusion, and (5) emerging trends.

**Figure 1:**
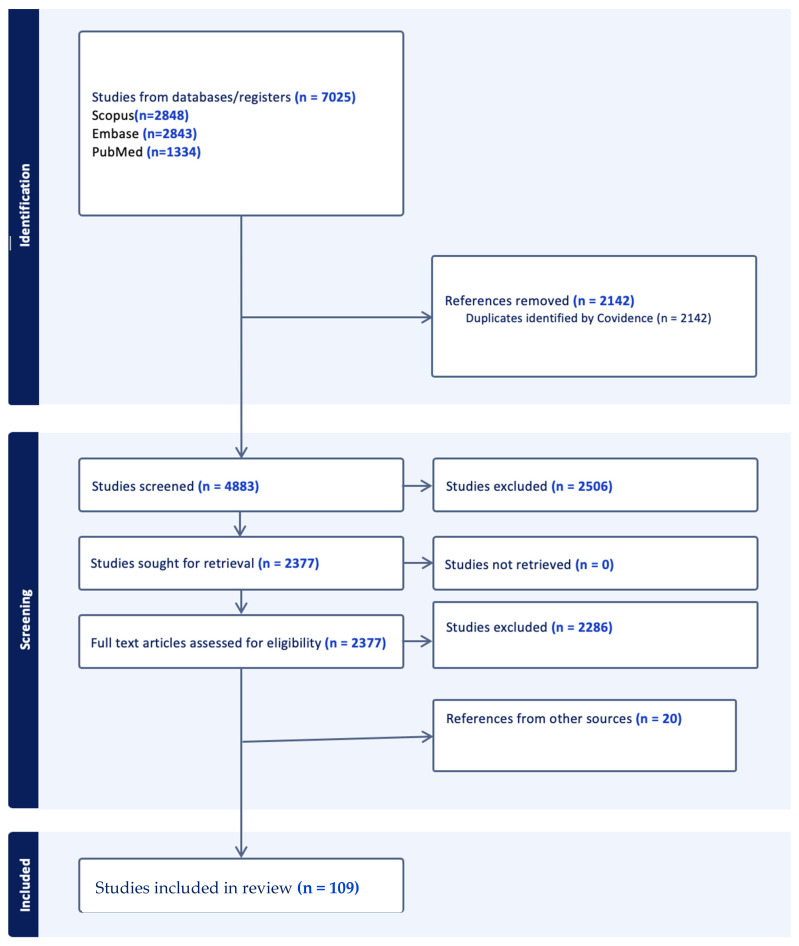
The workflow of search and selection. The search was conducted in February of 2025.

A PRISMA-style flow diagram that summarizes the literature search and selection process is shown in Figure 1.

## 3. Results

We identified and reviewed 109 peer-reviewed studies to support this study’s background and analytical framework. Our core analysis focused on studies published between January 2020 and January 2025 on artificial intelligence (AI) applications in PET and MRI neuroimaging for Alzheimer’s disease (AD). We categorized the studies into five core diagnostic domains:

- Image preprocessing and segmentation,
- Diagnosis and classification,
- Prediction and prognosis,
- Multimodal data fusion,
- Emerging trends in AI modeling.

Some representative studies from each category are presented in the following table (Table 1), along with a brief explanation of the primary objectives for each domain and common AI tools applied to address them.

**Figure 2:**
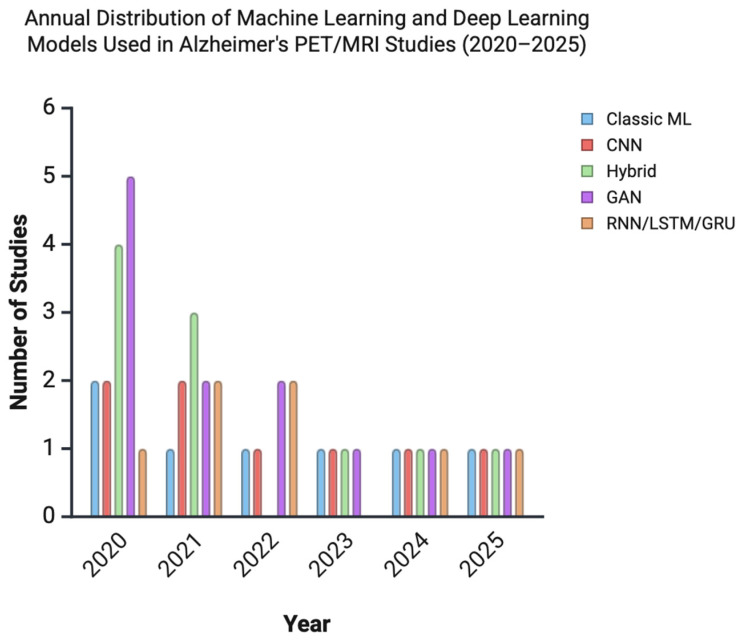
Bar chart illustrating the annual distribution of ML and DL model types used in Alzheimer’s disease PET/MRI studies included in this review, from 2020 to 2025. In 2020 generative adversarial networks (GANs) and hybrid models were most prominent. By 2023–2025, the relatively equal distribution across technologies suggests a stabilization in modeling strategies.

Deep learning approaches, particularly convolutional neural networks (CNNs), recurrent neural networks (RNNs), and generative adversarial networks (GANs), were the most frequently employed models across all domains. To illustrate these trends, we constructed a bar chart showing the distribution of AI models used in Alzheimer’s disease imaging studies included in our review article, from 2020 to 2025 (Figure 2). Several studies reported classification accuracies exceeding 95% for distinguishing AD from mild cognitive impairment (MCI) or cognitively normal (CN) individuals. These results must be interpreted cautiously due to potential risks of overfitting, lack of external validation, small sample sizes, and inconsistencies in imaging protocols.

**Figure 3:**
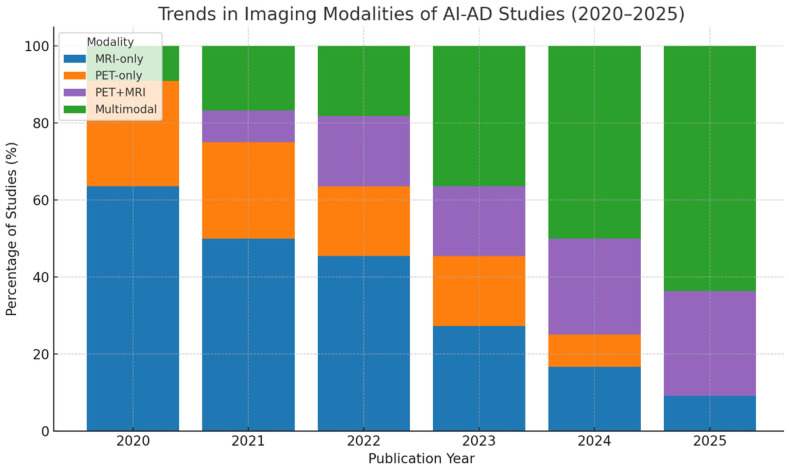
Modality trends in AI-based AD imaging studies from 2020 to 2025. This bar chart demonstrates the proportion of the studies utilizing MRI-only, PET-only, combined PET + MRI, and broader multimodal approaches (e.g., PET + MRI + clinical or CSF biomarkers). A progressive shift toward multimodal integration is evident, particularly after 2022, reflecting increased emphasis on combining structural, metabolic, and clinical information in predictive modeling.

Multimodal integration strategies, which combined MRI and PET imaging with clinical or neuropsychological data, generally demonstrated superior performance over unimodal models, particularly for early-stage diagnosis and longitudinal disease progression assessment. (Figure 3) Despite this, few studies conducted head-to-head model comparisons or reported confidence intervals, limiting direct comparability.

**Figure 4:**
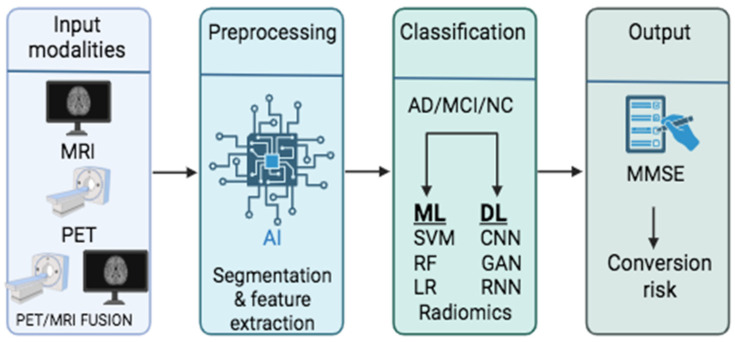
The AI pipeline in AD neuroimaging is presented in this figure. Preprocessing, including segmentation and feature extraction, is critical for forming raw MRI/PET data into meaningful inputs for further analysis using ML and DL tools. Accurate classification supports clinical outcomes by predicting cognitive decline (MMSE) and estimating conversion risk.

In parallel, methodological variability in image preprocessing pipelines further complicates cross-study comparisons, as differences in preprocessing steps such as skull stripping, noise reduction, and registration can significantly influence downstream model performance and generalizability. Some of these processes are necessary to make input modalities compatible with AI tools, thereby enabling the generation of interpretable results (Figure 4). Of the 109 studies included in this review, 46 (41.4%) were primary research articles that explicitly reported at least one image preprocessing step. Among these, 31 studies conducted skull stripping, 21 applied noise reduction (e.g., Gaussian filtering or non-local means denoising), and 24 performed image registration to standard anatomical templates such as MNI152. Bias field correction and intensity normalization were documented in 18 and 17 studies, respectively. These steps were typically implemented using standard neuroimaging software (e.g., FSL, SPM12, FreeSurfer) or deep learning-based preprocessing tools. The remaining studies were either reviews or papers that did not specify preprocessing details, often due to the use of preprocessed datasets like ADNI. Inconsistencies in imaging preprocessing pipelines emphasize the need for standardized and transparent reporting of preprocessing protocols to ensure reproducibility and facilitate model deployment.

Among these challenges, recent studies have focused on enhancing model interpretability, efficiency, and clinical applicability. Approaches such as explainable AI (XAI), contrast-agent-free imaging pipelines, and generative techniques for synthetic data augmentation are gaining traction. These innovations represent promising steps toward developing interpretable, scalable, and clinically viable AI tools for AD neuroimaging.

## 4. Discussion

### 4.1. Image Preprocessing and Segmentation

Image preprocessing and segmentation are foundational to AI-driven Alzheimer’s disease (AD) imaging pipelines (Figure 2). These steps are critical for reducing inter-scan variability and optimizing model performance across diverse imaging sources.

Preprocessing typically includes bias field correction, intensity normalization, and resampling to standardized voxel dimensions. These procedures help mitigate scanner-related artifacts and patient-specific noise while ensuring anatomical consistency across datasets [28,29,30]. Skull stripping, performed using tools such as the Brain Surface Extractor or FSL’s Brain Extraction Tool, removes non-brain tissues (e.g., scalp and orbits) and improves focus on cerebral structures, enhancing segmentation accuracy [28].

Noise reduction, significant for PET-MRI fusion, is commonly achieved with Gaussian filters or advanced denoising algorithms like CONN-NLM, improving structural integrity for multimodal image alignment [31]. Image registration to standard anatomical templates (e.g., MNI152) via software such as SPM12, FSL, or DARTEL facilitates cross-subject comparisons and region-of-interest (ROI) analyses, particularly in voxel-based morphometry or atlas-guided studies [30].

Traditional atlas-based segmentation methods (e.g., FreeSurfer, FSL-FIRST) have shown value in delineating regions like the hippocampus, amygdala, and ventricles. However, their limited adaptability to inter-individual anatomical variability has led to a shift toward deep learning-based segmentation, which offers superior spatial precision and robustness [32].

Recent studies employing architectures such as U-Net, V-Net, and nnU-Net have demonstrated high segmentation fidelity, with Dice similarity coefficients exceeding 0.90 for hippocampal and whole-brain tissue segmentation. Patch-wise CNNs (e.g., M-Net, hybrid multiscale models) enhance boundary detection by operating on subregions, reducing overfitting while preserving local context, especially practical in hippocampal segmentation from structural or multimodal inputs [18,33].

For PET segmentation, which faces challenges due to low anatomical resolution, deep conditional generative adversarial networks (cGANs) have shown promise. One model, FSPET, successfully segmented frontal lobe structures from FDG-PET by integrating anatomical priors and convolutional autoencoders, outperforming traditional methods in robustness and spatial accuracy [19,34].

Radiomics-based segmentation remains influential, particularly in hippocampal analysis. Studies have demonstrated that radiomic features extracted from T1-weighted MRI correlate with clinical markers (e.g., MMSE, amyloid-β, pTau), enabling high diagnostic accuracy. One investigation achieved an AUC of 0.961 using a random forest classifier to distinguish cognitively normal individuals from those with MCI or AD [18,34].

Functional imaging pipelines—especially those involving resting-state fMRI—employ preprocessing steps such as motion correction, slice timing adjustment, and spatial smoothing using SPM12 [35]. Subsequent segmentation with hybrid models, including 3D CNNs and LSTMs, enabled the discrimination of early MCI versus advanced AD, achieving classification accuracies above 96% by leveraging both spatial and temporal patterns [36].

Finally, innovative reinforcement learning models, such as Q-learning agents, have been proposed to automate hippocampal localization without manual intervention or atlas dependency. These models achieved comparable performance to fully supervised CNNs, improving generalizability and reducing memory overhead [30,37].

### 4.2. Diagnosis and Classification

Upon finishing preprocessing and segmentation, the image is ready for analysis and interpretation. Artificial intelligence has provided various tools utilized by researchers in the field of AD research, enabling a more efficient and accurate process for disease diagnosis and enhancing the classification procedure. Machine learning models have been widely used in improving the diagnostic accuracy in AD, with diverse efficacy and reproducibility. The more recent application of AI in medical imaging is deep learning. This model comprises an artificial neural network (ANN) that resembles human intelligence for complex input information processing and data analysis. Advanced techniques have been developed, leveraging the technology of DL, identifying atrophy patterns in MRI and hypometabolic networks, or specific biomarker detection in PET. AI has been further implemented in fMRI scanning and PET-MRI fusion, enabling a more complex processing of functional and anatomical input information without obscure patterns.

This section reviews and presents the most frequently used AI models for Alzheimer’s diagnosis and the classification of AD, mild cognitive impairment (MCI), and healthy controls. Table 1 summarizes the main diagnostic categories of AI use in AD imaging. The imaging modalities employed include MRI (structural MRI or functional MRI), PET (using FDG, amyloid-β, or tau tracers), and PET/MRI fusion models.

#### 4.2.1. Machine Learning in AD Diagnosis and Classification

Support vector machine (SVM) is a supervised machine learning tool that solves complicated tasks by setting variables and detecting patterns depending on the expected output (or labeled imaging when referring to neuroimaging) provided for the algorithm’s training [38]. SVM was widely applied to classify AD and MCI. Zhang et al. in 2021 [39] developed a modular-LASSO feature selection (MLFS) approach for AD/MCI classification, which incorporates the modularity of Fuzzy Bayesian Networks to detect discrete AD/MCI features. These features were then selected by the LASSO tool, classified via SVM, and applied to resting-state fMRI to detect AD/MCI [39]. SVM has also been employed in radiomics-based feature classification studies, yielding promising results [40,41]. For instance, Jiao et al. proposed a computational model based on SVM to identify radiomics signatures of tau tracer PET images, demonstrating higher accuracy than SUVR (84.8 ± 4.5% and 73.1 ± 3.6%) [40]. In a 2022 study by Nuvoli et al., differential diagnosis of AD with MCI based on hypometabolism of the temporal lobe in FDG PET imaging was conducted, showing a 76.23% accuracy of the linear SVM model [42]. A different approach has been proposed by Akramifard et al. in 2020 [43], where they improved the classification performance between AD/MCI and standard controls processed in an SVM classifier. Instead of expanding the sample size, the analysis focused on a smaller dataset by repeating key features within the input vectors- a method known as emphasis learning- achieving a classification accuracy of 98.81% between AD and NC [43]. Combination of SVM with graph measuring in functional MRI analysis, conducted by Wang et al. in 2023, showed an improved detection of AD, with maximum accuracy of 96.80% achieved in AD vs. health controls [44]. These high performances are likely attributed to the more precise separation of the most distinct diagnostic groups (AD vs. HC). In contrast, slightly lower accuracies were observed in identifying more intermediate stages, such as early to late MCI. Another graph–based fMRI study aimed at classifying AD, MCI, and healthy controls, combined SVM with LASSO feature selection technique, and showed high classification accuracy [45]. All the models in this section demonstrate impressive accuracy rates regarding either AD classification or AD/NC differentiation, outperforming more traditional technologies [40].

Logistic regression (LR) is an ML tool that uses a sigmoid-shaped curve to explore the correlation of the input with the probability of a specific output [46]. This feature was utilized by Van Loon et al. in 2022, where their team introduced the StaPLR (stacked penalized logistic regression) method, for automatic selection of the most significant views of sMRI, Diffusion Weighted Imaging (DWI) MRI, and fMRI for AD classification, providing a mean AUC of 0.942 with the 3-level StaPLR (hierarchical) compared to a mean AUC of 0.848 with the elastic net regression [47].

Decision tree (DT) and its extended form, random forest (RF), are supervised ML tools that classify data using roots that perform tasks divided into categories. RF accomplishes this task by producing multiple DTs that predict the output of a class [45]. Various studies have developed an RF model for AD classification [34,48,49,50]. A fusion study of Aβ PET and sMRI by Bao YW et al. in 2024 showed an improved AD classification when combining the two modalities to train the random forest model (AUC 0.89 and AUC of 0.71) [49]. RF has also been implicated in the feature selection procedure, combined with SVM, for AD classification based on statistics and volumetry. Keles et al. employed RF as the classifier; hence, all tools applied (BABC, BPSO, BGWO, and BDE) accomplished their highest accuracy, 0.863, 0.892, 0.905, and 0.893, respectively [51]. A fascinating investigation by Song et al., 2021 [50] compared RF, SVM, Multi-layer perception, and convolutional neural networks (CNN) for AD classification and biomarker detection using 63, 29, and 22 features. All three models demonstrated high accuracy in 63 features. RF, however, had the least reduction in accuracy percentage when 22 features were applied (−3.8% compared to −4% and −7.0% in MLP and CN, respectively). These findings highlight random forest’s consistent robustness across feature sets and modalities, making it a competitive and reliable classifier for Alzheimer’s disease, especially in comparison to other models like SVM, MLP, and CNN [50].

#### 4.2.2. Deep Learning in AD Diagnosis and Classification

Regarding deep learning tools, the most referenced techniques in the articles reviewed were convolutional neural networks (CNNs), recurrent neural networks (RNNs), autoencoders, and generative adversarial networks (GANs)—most of the studies aimed for AD diagnosis and classification employing CNN-based tools.

CNN analyzes the input data structured in a series of arrays (for instance, medical images), forming a complex network of interconnected layers (input, hidden, and output layers). These layers apply convolutional filters, small matrices that slide across the input to detect meaningful patterns like edges or textures, enabling the model to extract and learn relevant features for classification [52]. Multiple studies have implicated CNN models in detecting AD from multimodal imaging, achieving high accuracy. Table 2 presents a selection of top-performing AI models reported in high-impact studies. To ensure the conciseness of the section, the most frequently used CNN tools for diagnosis and classification will be subdivided and reviewed separately.

Visual Geometry Group Network (VGGNet) is a deep CNN tool widely implicated in AD research, [20,58,59], as it reduces the error rate by introducing fewer kernel features and increased network depth [60]. Kim et al., 2023 [20] proposed a highly accurate model by combining VGGNet with a 1D convolutional neural network software that extracts information about the brain’s contour, particularly the boundaries and shape patterns of cortical and subcortical regions. Incorporating VGGNet enhanced the existing model’s performance (accuracy of 0.986), allowing a more precise AD classification by measuring the shape of the patient’s brain, outperforming traditional tools like VGG-16, 19, and AlexNet in precision and accuracy. The highest accuracy and precision values were achieved in a 256 × 256 input size [20]. An additional study conducted by Mujahid et al. in 2023 developed a highly accurate ensemble model by combining VGG-16 with EfficientNet-B2, achieving significant improvements in early AD diagnosis [58].

ResNet is a distinct CNN model with multiple layers that excels at classifying inputs by introducing residual connections, reducing computational complexity [60,61]. ResNet has increasingly gained attention in AD classification and early disease detection, with a variety of ResNet models being used [21,26,53,62,63,64,65]. An illustrative case is the study of Odusami et al. in 2021, where a ResNet18 model was employed for AD classification in fMRI, achieving strong performance and high accuracy levels (99.99% accuracy) in differentiating early MCI from AD [21].

DenseNet is a CNN model introduced to maximize information flow between layers by employing dense feed–forward connections. Each layer receives the concatenated feature maps from preceding layers [60]. DenseNet model has been utilized for feature extraction, automating the procedure of AD diagnosis [53,66]. A comparative study of deep CNN models, in particular DenseNet, ResNet, and EfficientNet, was conducted by Carcagnì et al., demonstrating a better performance of very deep ResNet and DenseNet than the shallow versions of VGG and ResNet, with a 7% increase in the accuracy rate regarding the detection of AD in MRI [67]. A valuable research study by Sharma et al. [54] investigated the performance of a Hybrid AI model for AD diagnosis. They integrated a deep learning technique known as transfer learning, employing DenseNet-121 and DenseNet-201 for feature extraction, combined with machine learning classifiers, achieving an accuracy of 91.75% and specificity of 96.5% [54].

Other than VGG, DenseNet and ResNet, various CNN models have been developed and contributed to the research regarding the diagnosis and classification of AD [14,29,41,57,68,69,70,71,72]. For instance, a Dementia Network (DemNet) tool was implicated in AD staging instead of MRI, with an accuracy of 95.23% and AUC 0.97 [57]. AlzheimerNet has also been utilized as a fine—tuned classifier, achieving high accuracy and outperforming other traditional AD classifying tools [68]. LeNet is one of the first series of CNN architectures that incorporates MaxPooling layers to reduce data complexity by discarding elements that hold low values [73]. Hazarika et al. modified and applied this model in 2021 for AD classification, yielding a 96.64% performance rate [69].

Unlike CNNs, recurrent neural networks (RNNs) are specifically designed to capture temporal dependencies in data, enabling practical sequential analysis and prediction of time-dependent variables [73]. In 2024, Mahim et al. integrated gated RNNs with a vision transformer (ViT), leveraging the capability of gated RNNs to enhance current image processing by incorporating information from previously analyzed data [74,75]. The study demonstrated a high performance of the merged technique (99.69% for binary classification) for detecting and classifying AD from MRI.

Autoencoders are considered unsupervised learning tools that efficiently condense the information in an image and then recover the input data while maintaining its core attributes [76]. Al-Otaibi et al. in 2024, presented a dual–attention convolutional autoencoder technique, which demonstrated increased accuracy (99.02%) in real–time AD recognition, using MRI features [77]. A study based on fMRI employed a DL tool for efficiently discriminating normal aging from AD progression, demonstrating a specialized autoencoder network with excellent performance [78].

GAN has contributed to medical image processing, synchronizing new image output with two neural networks after training. This tool allows the generation of new data and adaptation to shifting domains, as one network generates the image, while the other separates the features [79]. A study published in 2023 introduced a Loop–BasedGAN for Brain Network (BNLoop-GAN) model, which aimed to uncover the distribution of the underlying brain networks by using a set of different tools, including conditional generation. Successful discrimination was achieved using resting-state fMRI and structural MRI (sMRI) between healthy controls and AD patients with a sensitivity and specificity of 81.8% and 84.9%, respectively, on multimodal brain networks, outperforming other tested models [55]. An alternative application of generative adversarial networks in MRI was demonstrated by Chui et al., where CNN and transfer learning (TL) were introduced to improve classification accuracy and incorporate data from various datasets. GANs were subsequently used to augment less frequently occurring data, enabling the model to achieve higher accuracy in detecting AD [80].

### 4.3. Prediction/Prognosis

AI’s contribution to Alzheimer’s research is evolving beyond disease diagnosis, playing a growing role in early detection and accurate prognosis of disease progression. Various AI models and novel techniques have been developed to precisely detect MRI/PET biomarkers for accurate disease tracking. Table 3 summarizes the pathological features and associated biomarkers commonly analyzed in AI-driven Alzheimer’s disease studies. Longitudinal studies have enriched this area by enabling models to track volumetric and metabolic changes in key regions using MRI and PET, respectively, while integrating these modalities with molecular and clinical data. AI-driven techniques, such as linear mixed-effects analysis, facilitate this integration and analysis [81]. Temporal observations of disease progression through fusion of imaging alterations with clinical cognitive scales allow a more comprehensive view of AD.

The importance of predicting Alzheimer’s disease forecasts has been the focus in recent years, as the capability of detecting early signs of progression of the disease could be potentially life–changing for the patients, enabling an earlier intervention. A recurrent neural network (RNN) model that uses long short-term memory (LSTM) was utilized by Aqeel et al. in 2022 to predict neuropsychological and MRI biomarkers in the progression of time, aiming to distinguish between AD and MCI, based on the predictions [22]. Khalid et al. designed a feed-forward neural network technology that combines aspects from GoogLeNet and Dense–121, to detect Alzheimer’s disease and model the progression trajectory. The accuracy achieved was 99.7%, with an AUC of 0.99 [23]. Two different research studies proposed deep learning tools that targeted the classification of MRI images (sMRI and fMRI) to identify dementia and classify it into stages depending on the severity [36,84]. Both studies revealed comparable results, achieving more than 80% accuracy, with Noh J.’s [36] study stating the need for a more generalized testing of their proposed tool.

Several AI tools have been developed and extensively explored for predicting the development of AD from mild cognitive impairment (MCI) [85,86,87,88]. A radiomics-based feature study on PET was conducted by Peng et al. in 2023 [85], exploring the role of white matter as a predictive component for MCI to AD progression. This study integrated PET-derived radiomics features with clinical assessment scales, for instance, Clinical Dementia Rating (CDR) and Alzheimer’s Disease Assessment Scale (ADAS), and by applying multivariate logistic regression (ML tool), achieved high sensitivity and specificity in predicting the progression of MCI to AD, 87% and 78%, respectively, and a ratio hazard computed when the model was clinically evaluated with 95% confidence interval [85]. An additional investigation of MCI to AD progression was demonstrated by Lin et al., where an extreme learning machine (ELM) tool for grading five different modalities was utilized, yielding excellent performance in disease progression prediction [86]. A different approach for detecting the MCI to Alzheimer’s dementia conversion has been proposed by Fakoya et al. in 2024 [87], aiming to overcome two main barriers: the complexity in data processing of 3D MRI and PET scans, and the combination of both modalities in a way that retains their individual visual information. This study demonstrated a CNN model with high accuracy (94.0%) by integrating slices from both MRI and PET scans, preserving the unique features of each modality while reducing processing time due to the model’s streamlined architecture [87].

Various studies have investigated the AD progression based on imaging biomarkers. Pan et al. in 2023 [56] developed a DL technique called Ensemble 3DCNN, which provides insights into the widespread structural alterations in the brain during AD progression. The model generates a score based on the detected alterations in different regions in MRI scans [56]. The trajectory of lesion progression in Alzheimer’s patients has been investigated by Kim et al. in 2021 [89]. They developed a network using an autoencoder to initially condense MR images, obtained from various AD stages, to latent vectors, and then predict the latent vector of an image at a target time point [89]. Crystal et al. [90] utilized already existing brain age predicting models to develop an ML technology that predicts the age of healthy individuals based on imaging features from the FLAIR sequence. Then they illustrated the predicted–actual age difference with an estimation called BrainAGE, which was then applied as a marker for the longitudinal analysis [90].

Amyloid-beta plaques and tau tangles are key hallmarks of Alzheimer’s disease (AD), often used as biomarkers in AI models. These pathological changes are excessively implicated in medical imaging, especially for the development of tools that assist in the prediction of Alzheimer’s disease progression. A linear support vector regression with multiple variables has been developed by Wang et al. in 2024, which uses tau tracer-based PET images to predict the brain age of healthy individuals [83]. A study by Alongi et al. demonstrated a radiomics analysis of features extracted from 18-FDG PET images with an ML tool to predict AD occurrence. Clinical assessments and amyloid-based PET scans were conducted to compare the results [82]. Alternatively, the aim of Chattopadhyay et al. in the 2024 [91] study was to predict the existence of Aβ plaques, using T1-WI MR images. Multiple DL models have been developed and examined, demonstrating a promising approach for the prediction of Alzheimer’s pathology in MCI patients [91].

In recent years, the application of AI to predict cognitive scores from MRI data has been increasingly explored and refined. Habuza et al., 2022 [92] proposed a convolutional neural network model based on regression technology to predict standard controls’ cognition level using MR images. This approach was subsequently applied in MCI patients, revealing a significant divergence between the normative aging model and cognitively impaired patients. The model discriminates precisely between normal individuals and MCI patients with an AUC of 99.57% [92]. Liang et al. conducted a relevant study, utilizing a multi-task learning (MTL) tool for the prediction of cognitive status, based on structural association [93].

### 4.4. Multimodal Fusion and Clinical Integration

**Figure 5:**
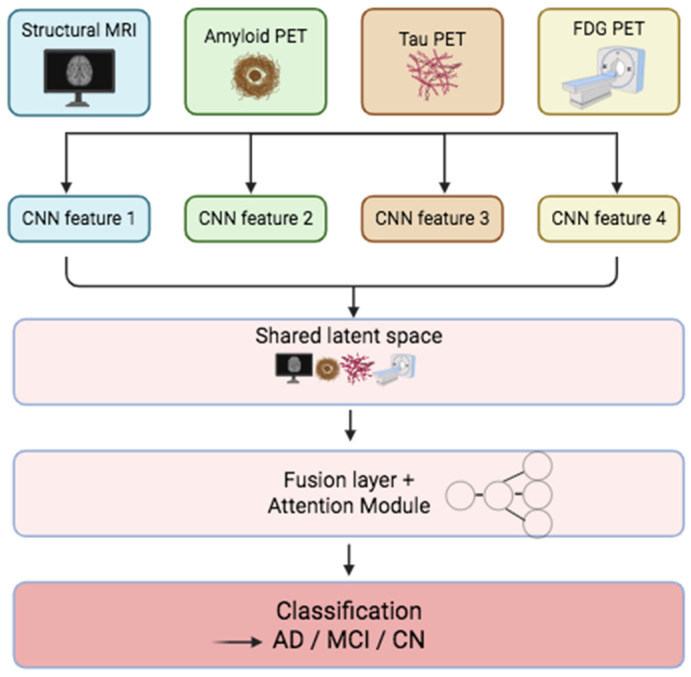
This scheme demonstrates the multimodal Fusion Architecture. Multimodal inputs (MRI, amyloid, tau, and FDG PET) are processed through CNNs to extract features, which are integrated into a shared latent space. A fusion layer with an attention module enhances accurate classification and CN learning.

Multimodal fusion in AD diagnosis combines data from multiple sources such as MRI, PET, cerebrospinal fluid (CSF) biomarkers, and cognitive scores to improve diagnostic accuracy, staging, and prognosis [24] (Figure 5). Each modality analyzes different biological substrates of AD, with MRI providing detailed information on anatomy and FDG-PET reflecting glucose metabolism [25]. In addition, amyloid and tau PET visualize pathological deposition patterns. The complementary nature of these modalities frequently outperforms unimodal models, especially in detecting early stages of dementia and predicting disease progression [94,95].

However, such early fusion approaches may introduce redundancy and imbalance, particularly when the combined modalities differ in resolution, scale, or feature count. These disparities can lead to the dominance of one modality or the dilution of critical information. Zhang et al. proposed a discrete cosine transform (DCT)-based convolutional sparse representation framework to address these limitations, extracting compact and informative spatial-frequency features from MRI and PET before fusion [96].

By contrast, middle fusion techniques integrate information at deeper network stages, after each modality undergoes separate feature extraction. This design helps preserve richer intermodal interactions and minimizes interference during early processing. Kim et al. [24] demonstrated this approach using a dual-path CNN with shared-weight convolutions and depth-wise separable blocks to process FDG-PET, amyloid PET, tau PET, and structural MR. Their architecture merged features in a shared latent space. It achieved high-balanced accuracies of 100% for AD vs. CN and 76% for MCI vs. CN, and robust performance for stable vs. progressive MCI [24].

Some studies applied multi-kernel learning and ensemble classifiers to combine imaging with clinical variables such as MMSE, CDR, and CSF biomarkers. Chiu et al. employed support vector machines using composite features from imaging, cognitive assessments, and demographics to differentiate SCD, MCI, and AD, achieving classification [25]. The classification accuracy findings underscore the clinical utility of integrating structured patient data with imaging features for early-stage detection.

Attention-based fusion models have also emerged to emphasize intermodal relationships. For instance, Huang et al. designed a voxel-wise correlation matrix between MRI and FDG-PET to guide an attention module that fused metabolic and structural signals, maximizing AD classification performance [97]. Similarly, multi-branch neural networks with shared and modality-specific pathways have integrated imaging and cognitive data to improve stage-specific prediction across the AD spectrum [95].

Region-of-interest (ROI)-based fusion also remains popular, targeting AD-susceptible areas such as the hippocampus, entorhinal cortex, and cingulate cortex. This strategy improves model interpretability and reduces computational burden. Several studies have used CNN-based multi-atlas segmentation to restrict analysis to these critical ROIs [24,97,98].

From a clinical standpoint, several proposed models have been validated using large-scale public datasets such as ADNI and OASIS. Some studies have even incorporated real-world data from memory clinics [59,94,96], demonstrating generalizability and clinical relevance. Lightweight architectures like extreme learning machines (ELMs) and attention-guided multi-branch CNNs have shown promise for integration into fast-paced clinical workflows [96,99].

Despite these advancements, widespread clinical adoption is hindered by cross-center heterogeneity, inconsistent acquisition protocols, and a lack of standardized model pipelines. Recent studies have introduced domain adaptation layers, modality-specific normalization, and attention-based calibration blocks to overcome these challenges, improve reproducibility, and reduce bias [59,95,100].

Explainability remains another primary focus. Visual tools such as saliency maps and Grad-CAM are increasingly embedded within multimodal frameworks to provide transparency and foster clinician trust in AI-based decision support systems [1,99,101].

### 4.5. Emerging Trends and Future Directions

**Figure 6:**
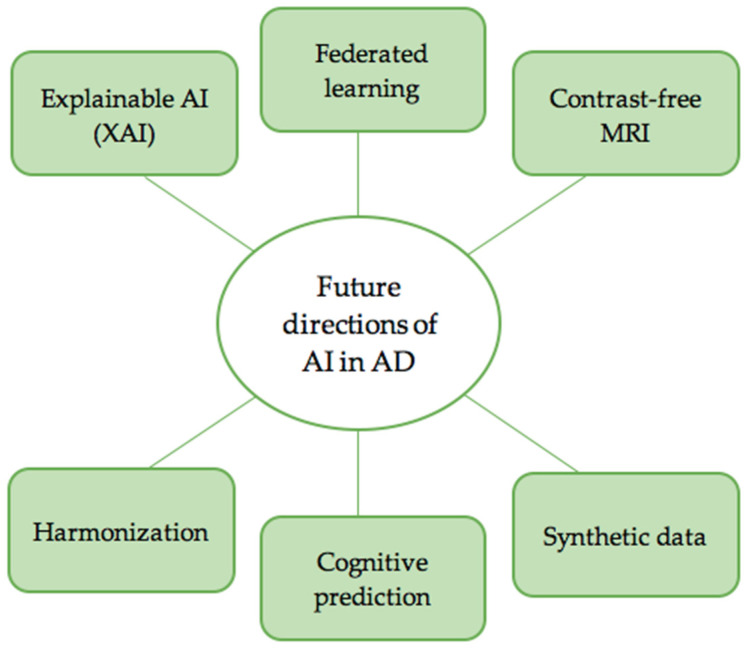
Emerging directions in AI for Alzheimer’s disease include explainable AI (XAI), contrast-free imaging, federated learning, harmonization, cognitive prediction, and the use of synthetic data via GANs, all of which aim to enhance clinical applicability, generalizability, and interpretability.

Emerging trends in AI for AD neuroimaging are focusing on making AI models more applicable, interpretable, and clinically useful. As more neuroimaging data is gathered, AI’s role in simplifying analysis and drawing meaningful conclusions is becoming more critical (Figure 6).

One of the main challenges facing AI in healthcare settings is the “black box” nature of most deep learning algorithms. Explainable AI (XAI) is, therefore, a fundamental research area. Amoroso et al., in 2023, emphasize the importance of XAI, showing that Shapley values by fairly distributing the contribution of each feature to a model’s output and evaluating how each one impacts predictions across all possible feature combinations can help us understand how AI models make their predictions [27]. This added transparency is crucial for building trust with clinicians and researchers, as it allows them to identify which brain regions and imaging features are most significant in characterizing AD. XAI helps to not only validate AI’s findings but also uncover new insights into the underlying mechanisms of the disease. Among the 46 primary studies analyzed in this review, 8 explicitly integrated explainability mechanisms into their model architectures [21,50,53,69,71,75,84,102]. These additions improve model transparency and accountability, critical for clinical translation, regulatory approval, and clinician trust in AI-assisted decision-making processes.

Another important initiative is reducing the dependence on contrast agents in MRI scans. One study showed a deep learning model that could produce contrast-equivalent information from non-contrast MRI [26]. This breakthrough highlights safety issues linked to contrast agents, particularly regarding gadolinium retention in the brain after repeated MRI use, as noted by regulatory agencies, as well as the rising costs and complexities of imaging procedures. AI facilitates readily available non-contrast MRI and enhances the accessibility and potential clinical benefits of advanced neuroimaging analysis.

Higher efficiency and standardization in Alzheimer’s disease quantification are also needed. Yamao et al., in 2024, developed a deep learning method to automate the computation of the Centiloid scale from amyloid PET images [102]. The Centiloid scale is key in standardizing amyloid quantification across studies, imaging radiotracers, and equipment. By reducing manual effort and variability, the automated approach enhances the reproducibility and practicality of Centiloid-based analysis in clinical and research settings.

Generative models are also proving to be valuable in AD neuroimaging. A recent study explored various generative models for synthetic MRI data, highlighting their potential to enhance limited datasets [103]. Similarly, another study showcased how super-resolved structural MRI can positively impact the detection of mild cognitive impairment [104]. These abilities to improve image resolution or generate synthetic data decrease the problem of limited data in medical imaging, which ultimately enhances the reliability and applicability of AI models.

In parallel, generative adversarial networks (GANs) have emerged as powerful tools in positron emission tomography (PET) imaging. GAN-based frameworks have been developed to perform super-resolution reconstruction, denoising of low-dose PET scans, and even cross-modality synthesis, generating pseudo-PET images from MRI or CT inputs. Such applications are particularly valuable in neurodegenerative diseases, where concerns about frequent imaging and radiation exposure make non-invasive, low-dose, or synthetic imaging alternatives especially important. For instance, GANs can simulate standard-dose PET scans from ultra-low-dose acquisitions, reducing patient exposure without compromising diagnostic utility. Additionally, cross-domain synthesis methods leveraging GANs offer new opportunities in multimodal integration, improving lesion detectability and disease staging in Alzheimer’s disease and related dementias [105]. These innovations support the goal of making advanced imaging more accessible, cost-effective, and clinically applicable across resource-limited settings.

Another critical factor is addressing the variability in data collection across different locations and timeframes. A study in 2024 showed that deep learning techniques could reduce the time of harmonizing PET/MR data from various scanners [106]. This is vital for longitudinal studies, multi-center collaborations, and early-phase clinical trials evaluating diagnostic or therapeutic agents. Harmonization ensures that imaging biomarkers remain consistent and comparable across different platforms, enhancing the reliability of AI-based analyses and enabling more accurate interpretation of treatment effects and disease progression in heterogeneous datasets.

Finally, Liu et al. (2022) significantly improved the development of a recognition network that combines a spatial transformation attention mechanism with multiple phantom convolutions [26]. These network architecture advancements enhance AI’s capacity to detect small structural changes in MRI images linked to AD.

#### 4.5.1. Clinical Importance

The role of artificial intelligence in diagnosing, classifying, and predicting AD has been extensively examined, highlighting the robustness and high accuracy levels of these techniques. From a clinician’s perspective, these technological advancements, particularly machine learning (ML) and deep learning (DL) in modeling Alzheimer’s disease trajectory, will enable radiologists to interpret extensive, complex imaging data with greater precision and efficiency. As a result, diagnostic accuracy will improve, reducing misdiagnosed dementia cases and enhancing workflow in daily clinical practice. The integration of multi-modal data is also of high clinical importance in Alzheimer’s disease classification. MRI and PET scans, fluid biomarkers, and clinical assessment scales provide valuable information regarding the disease progression. Thus, CNN models [24] and other ML/DL tools [25,97] facilitate this data fusion, supporting a more holistic approach to clinical decision-making. An alternative application of AI tools in clinical and research settings is training medical residents and research interns in neuroimaging in neurodegenerative diseases. This foundational education ensures a new generation of scientists and clinicians is equipped to contribute effectively to the evolving field of neuroimaging.

However, some key issues must be resolved before AI models can be incorporated into clinical practice. A significant consideration involves ethical concerns, which are fundamental to the responsible deployment of AI in Alzheimer’s disease diagnosis. These include ensuring data privacy, maintaining patient autonomy, implementing robust data security, and obtaining informed consent for using personal imaging data in algorithmic analyses. Patients must understand how their data will be processed and retain control over its use in clinical and research contexts.

In parallel, regulatory oversight is evolving to address the unique challenges AI tools pose in healthcare. Agencies such as the U.S. Food and Drug Administration (FDA) and the European Medicines Agency (EMA) increasingly require transparency, algorithmic explainability, and rigorous clinical validation before these tools can be approved.

Finally, interpretability remains one of the most critical factors determining whether a model is suitable for integration into clinical workflows. Clinicians must be able to understand and trust the rationale behind AI-driven decisions. This need for transparency has driven the rise in explainable AI (XAI) as a central trend in developing diagnostic support tools.

#### 4.5.2. Limitations

This review has some limitations that are important to consider when interpreting its findings. First, differences in imaging protocols, sample sizes, and AI model architectures across the included studies make direct comparisons difficult. Many studies used small, single-center datasets and did not validate externally, which limits how well their results apply broadly. In some cases, performance metrics like AUC, sensitivity, and specificity were reported without confidence intervals or statistical comparisons, making these outcomes less clear and reproducible.

While some research showed diagnostic accuracy over 95% as seen in (Table 4), these results might be affected by overfitting, data leakage, or the lack of standardized validation procedures. The variety of cross-validation methods (e.g., k—fold, leave one out) and the absence of benchmarking against established clinical tools further hinder translating these models into clinical practice.

This review mainly focuses on PET and MRI modalities. It does not cover imaging techniques such as optical imaging, digital pathology, or non-imaging data such as genomics and fluid biomarkers, which could enhance imaging models in future applications.

Although key studies were thematically grouped and critically summarized, this review does not include a formal meta-analysis or systematic quality assessment. Though outside this review’s scope, these methods could provide more detailed comparative insights. For example, a recent meta-analysis of 18 studies using deep learning on MRI for AD/MCI reported a pooled sensitivity of 0.84, a specificity of 0.86, and an AUROC of 0.92 [107]. Another systematic review of 101 structural MRI studies showed significant variation based on dataset, model architecture, and validation strategy, highlighting the field’s diversity and the need for standardization [108]. A relevant meta-analysis conducted by Sun Y. et al. in 2025 [109] highlighted the high-performance metrics of DL tools for AD diagnosis using PET scan, with 36 studies included in their article, with most achieving a pooled AUC of 98%. The same study commented on the need for a more standardized procedure regarding the external validation and sample size employed for the tools’ evaluation to attain better robustness and reproducibility of the models [109].

Finally, the fast pace of AI development may make some findings quickly outdated. Many studies in this review predate the widespread use of advanced techniques like transformer models, federated learning, and self-supervised pretraining, which are likely to influence future AI models for AD imaging.

#### 4.5.3. Future Directions

Future research should tackle limitations in AI-driven Alzheimer’s disease neuroimaging studies by emphasizing clinical robustness, broader applicability, and strict methodological standards. Efforts should prioritize creating large, multicenter datasets with standardized imaging protocols to address the shortcomings of small-center cohorts. External validation using prospective or multi-institutional data ensures real-world relevance and minimizes overfitting. Consistent evaluation metrics, confidence intervals, and transparent reporting are essential for enhancing reproducibility. Moreover, AI techniques like transformer-based architectures, self-supervised learning, and federated learning offer potential for developing more scalable and privacy-conscious models. Incorporating non-imaging biomarkers, such as genomic and fluid-based data, could improve diagnostic precision and prognostic insights. Lastly, future work should focus on integrating AI tools into clinical practice, assessing their effects on workflow efficiency, clinical decision-making, and patient outcomes.

## 5. Conclusions

Artificial intelligence has significantly shaped the modern perspective on neuroimaging, with various applications of machine learning and deep learning integrated into radiographic analysis and interpretation of medical images. Initially, machine learning was introduced in neuroimaging as a tool to support the diagnostic process, offering precise and consistent aid in decision-making. As the term suggests, machine learning in imaging is a technology “trained” by humans to identify structures in MRI/PET, quantify the volume of regions of interest, and determine whether a pattern of atrophy or hypometabolism exists. ML can be supervised or unsupervised, depending on the model designed. The demand for a technology capable of generating meaningful output by providing raw input alone highlighted the potential for developing deep learning models, a subfield of machine learning inspired by the human brain, which processes data using artificial neural networks. These networks allow the system to autonomously analyze pre-processed data and generate outputs by forming hierarchical representations that support pattern recognition. Feed-forward learning allows DL models to enhance discriminative features while suppressing irrelevant information, key processes in their functioning.

Both ML and DL have been leveraged by neuroimaging across all image processing steps. They assist in processing and segmenting key brain areas, provide tools for precisely detecting disease-related patterns in the CNS, and enable classification into stages, contributing to constructing more reliable predictive models. These technologies have been extensively utilized in Alzheimer’s disease research, aiming to pave the way for novel diagnostic tools that allow a more rapid and accurate disease detection during the early stages, or even before the onset of clinical symptoms, thus facilitating a fast intervention.

Numerous models have been developed and presented by research teams, particularly following the FDA’s approval of Tauvid, that aim to disrupt the conventional trajectory of the disease. These techniques have proved high accuracy and excellent performance, each targeting a slightly different aspect of the disease. Although the future of neuroimaging with integrated tools appears promising, several key concerns have emerged. External validation is a significant aspect that defines the actual performance, as the model must be tested on a different dataset to prove its reproducibility and accuracy. Respecting and ensuring data privacy is another primary concern, as the tools require vast amounts of data to be trained and achieve consistent results.

The cutting-edge contributions in the field offer considerable potential in Alzheimer’s disease, not only for disease detection and management, but also for outlining future directions in research and clinical application. Despite the numerous considerations, a new “era” in neuroimaging was introduced, leading to a more personalized medicine, where diseases are managed depending on the features detected, classified into distinct stages by integrating imaging modalities with clinical scales, and heading towards accurate prognostic markers and predictive models. With continued interdisciplinary innovation, this progress brings us closer to earlier diagnoses, more effective interventions, and improved patient lives.

## Figures and Tables

**Table 1:** Diagnostic categories of AI use in AD imaging.

Domain	Description	Modalities	Common AI Methods	Example Studies
Preprocessing and Segmentation	Prepares data for modeling: skull stripping, noise correction, registration	MRI, PET	U-Net, nnU-Net, GANs, Radiomics	[18,19]
Diagnosis and Classification	Identifies disease stage or detects AD during early stages	MRI, PET	CNNs (ResNet, DenseNet, VGG), SVM, RF	[20,21]
Prediction and Prognosis	Forecasts disease progression, enables longitudinal analysis studies, and provides risk assessment	MRI, PET, fMRI	RNN, LSTM, Logistic Regression	[22,23]
Multimodal Fusion	Combines imaging output, blood/CSF biomarkers, and clinical data to obtain a comprehensive final, cumulative result	MRI + PET + MMSE + CSF	Ensemble CNNs, Dual-Path CNNs, SVM	[24,25]
Emerging Trends	New techniques like XAI (enhancing explainability), synthetic data (generating training input for AI models), and harmonization (enabling better standardization)	All	XAI, Generative Models, Transformers	[26,27]

**Table 2:** Top-performing AI models in high-impact studies.

AI Model	Task	Input Modality	Accuracy/AUC	Study
ResNet18	AD vs. MCI classification	fMRI	99.99%	[53]
DenseNet121 + SVM	AD Classification	T1 MRI	91.75%	[54]
BNLoop-GAN	Brain network generation	fMRI + sMRI	98%	[55]
Ensemble 3D CNN	Progression Tracking	Longitudinal MRI	--	[56]
DemNet	AD staging	MRI	95.23%	[57]

**Table 3:** Pathological features and imaging biomarkers in AI-driven AD studies.

Pathological Feature	Modality	AI Applications	Typical Output	References
Amyloid-β (Aβ)	PET (AV45, PiB)	Diagnosis, prognosis	SUVR, Centiloid scaling	[24,49,82]
Tau Tangles	PET (Tauvid)	Staging, prognosis	SUVR, cortical distribution	[24,40,83]
CSF Aβ42/tau ratio	Biochemical (CSF)	Risk prediction, multimodal fusion	The ratio “drop” correlates with AD conversion	[50]
Medial Temporal Atrophy	T1 MRI	Segmentation, classification, brain age	Volume loss in the hippocampus/entorhinal cortex	[34,51,63]
FDG Hypometabolism	FDG PET	Multimodal fusion, deep learning	Reduced glucose metabolism in parietal/temporal	[24,42]
White Matter Integrity	DTI/Structural MRI	Prediction of progression, subtype analysis	FA and MD abnormalities in frontal-parietal tracts	[35,36]

**Table 4:** Description of some remarkable AI models used in AD during 2020–2025.

Title	Author	Year	AI Model/Architecture	Modality	Dataset	Performance Metrics	Model Insights	Limitations	Reference
3D CNN Design for the Classification of Alzheimer’s Disease Using Brain MRI and PET.	Khagi B. et al.	2020	Encoder-based 3D CNN	MRI and/or PET	ADNI Baseline (BL) projects	Accuracy: 94.56%	Diverges receptive fields to optimize feature extraction efficiency	Dataset size and class imbalances (AD, MCI)	[29]
Diagnosis of Alzheimer’s Disease Using Convolutional Neural Network With Select Slices by Landmark on Hippocampus in MRI Images	Pusparani Y et al.	2023	Resnet50 and LeNet	MRI	ADNI	Accuracy: 98%	Attention model to improve accuracy	No external validation	[63]
3D CNN for AD detection using MRI	Haijing et al.	2021	ResNet	MRI	ADNI	Accuracy: 97.1%	Captures more information from MRI	Limited interpretability/No external Validation	[64]
Using a Patch-Wise M-Net Convolutional Neural Network for Tissue segmentation in brain MRI images	Yamanakkanavar N. et al.	2020	CNN M-Net	MRI	OASIS	Accuracy: 94.81–96.33%	Automatic segmentation of brain MRI scans	M-Net is prone to missing details in certain regions	[33]
An Intelligent System for Early Recognition of Alzheimer’s Disease Using Neuroimaging	Odusami et al.	2022	ResNet DesNet	MRI	ADNI	Accuracy: 98.86%	Grad class activation map	No external validation	[53]
Analysis of Features of Alzheimer’s Disease: Detection of Early Stage from Functional Brain Changes in Magnetic Resonance Images Using a Finetuned ResNet18 Network	Odusami et al.	2021	ResNet 18	fMRI	ADNI	Accuracy: 99.99%	Integrates structural and metabolic info	Overfitting	[21]
FDG-PET to T1 Weighted MRI Translation with 3D Elicit Generative Adversarial Network (E-GAN)	Bazangani F. et al.	2022	Elicit GAN	FDG PET	ADNI	Structural similarity (SSIM): 75%	FDG-PET to 3D T1-WI generation	Long training time and model tested only on healthy subjects	[19]
Brain MRI Analysis for Alzheimer’s Disease Diagnosis Using CNN-Based Feature Extraction and Machine Learning	Duaa AlSaeed	2022	ResNet 50	MRI	ADNI and MIRIAD	Accuracy: 85.87–99%	Efficient feature extraction	No external validation	[65]
Based on Tau PET Radiomics Analysis for the Classification of Alzheimer’s Disease and Mild Cognitive Impairment	Jiao F. et al.	2023	Radiomics analysis	Tau PET	ADNI	Accuracy: 84.8%	Prediction of tau positive MCI or ApoE ε4 presence	Not biopsy-confirmed AD diagnosis and external cohorts with small subject number employed	[40]
MPC-STANet: Alzheimer’s Disease Recognition Method Based on Multiple Phantom Convolution and Spatial Transformation Attention Mechanism	Yujian et al.	2022	ResNet50	MRI	Multiinstitutional	Accuracy: 96.25	Space conversion attention	No external validation	[26]
Functional Brain Network Measures for Alzheimer’s Disease Classification. IEEE Access.	Wang L et al.	2023	SVM-linear	fMRI	ADNI	Accuracy: 96.80 (HC vs. AD)	Identification of significantly altered networks between HC, MCI states and AD	Only 36 out of 360 regions taken into account using J-HCPMMP parcellation	[44]
Leveraging Brain MRI for Biomedical Alzheimer’s Disease Diagnosis Using Enhanced Manta Ray Foraging Optimization Based Deep Learning	R. Syed Jamalullah	2023	DesNet 121	MRI	ADNI	Accuracy: 98.29%	Enhanced Manta ray Foraging Optimization	No external validation	[66]
Convolution Neural Networks and Self-Attention Learners for Alzheimer Dementia Diagnosis from Brain MRI	Pierluigi Carcagnì et al.	2023	CNN	MRI	ADNI and OASIS	Accuracy: 77%	Self-attention learners	No external validation, information may be lost during feature extraction	[67]
Analyzing Hierarchical Multi-View MRI Data With StaPLR: An Application to Alzheimer’s Disease Classification	Van Loon. et al.	2022	Stacked penalized LR (STaPLR)	MRI (DWI, sMRI, fMRI)	PRODEM (Medical University of Graz)	Accuracy: 88.8%	MRI view selection most significant for disease prediction	Binary selection process: each view is either selected or not selected, which may differ from subject to subject	[47]
HTLML: Hybrid AI Based Model for Detection of Alzheimer’s Disease	Sarang et al.	2022	DesNet 121, DesNet 201	MRI	Kaggle	Accuracy: 91.75%	Hybrid architecture improves stability	Complex to reproduce, no external validation	[54]
DEMNET: A Deep Learning Model for Early Diagnosis of Alzheimer Diseases and Dementia From MR Images	Suriya et al.	2021	CNN	MRI	Kaggle ADNI- external validation	Accuracy: 95.23%	Multilayer architecture	Complex to reproduce	[57]
Combined Quantitative amyloid-β PET and Structural MRI Features Improve Alzheimer’s Disease Classification in Random Forest Model—A Multicenter Study	Bao Y. et al.	2024	RF	Aβ-PET, sMRI	AIBL database GAIN dataset	Accuracy: 81% (HC vs. AD)	Aβ PET features for AD detection using ML models	Demographical information missing from subjects, limited sample size	[49]

## Data Availability

No new data were created or analyzed in this study. Data sharing is not applicable. All data discussed are derived from publicly available, peer-reviewed publications cited within the manuscript.

## Abbreviations

AD	Alzheimer’s Disease
AI	Artificial Intelligence
AUC	Area Under the Curve
ANN	Artificial Neural Network
CNN	Convolutional Neural Network
CSF	Cerebrospinal Fluid
DL	Deep Learning
DT	Decision Tree
FDA	Food and Drug Administration
FDG	Fluorodeoxyglucose
GAN	Generative Adversarial Network
LLM	Large Language Model
LSTM	Long Short-Term Memory
ML	Machine Learning
MCI	Mild Cognitive Impairment
MRI	Magnetic Resonance Imaging
MMSE	Mini-Mental State Examination
PET	Positron Emission Tomography
RF	Random Forest
RNN	Recurrent Neural Network
ROI	Region of Interest
SVM	Support Vector Machine
SUVR	Standardized Uptake Value Ratio
XAI	Explainable Artificial Intelligence

## Appendix A

### Appendix A.1. Pubmed Search Strategy

Date Searched: 2 March 2025

**Set #**	**Concept**	**Syntax**	**Results**
1	AI	“artificial intelligence” [MeSH Terms] OR “Neural Networks, Computer” [Mesh] OR “Image Processing, Computer-Assisted” [Mesh] OR “Deep Learning” [mesh] OR “Machine Learning” [mesh] OR “Artificial Intelligence” [tw] OR “Artificial Neural Network” [tw] OR “Convolutional Neural Network” [tw] OR “Deep Learning” [tw] OR “Machine Learning” [tw]	606,889
2	PET/MRI	“Positron-Emission Tomography” [Mesh] OR “Magnetic Resonance Imaging” [Mesh] OR “Positron Emission Tomography” [tw] OR “PET” [tw] OR “Magnetic Resonance Imaging” [tw] OR MRI [tw]	921,043
3	Alzheimer	“Alzheimer Disease” [Mesh] OR Alzheimer [tw] OR Alzheimers [tw] OR Alzheimer’s [tw] OR “Senile Dementia” [tw] OR “Presenile Dementia” [tw]	227,267
4	Diagnosis or detection	“Diagnosis” [Mesh] OR “diagnosis” [Subheading] OR diagnoses [tw] OR diagnose [tw] OR diagnosis [tw] OR detect * [tw]	13,530,301
5	combining	#1 AND #2 AND #3	4124
6	filters	# 4 NOT (“animals” [MeSH Terms] NOT “humans” [MeSH Terms]) NOT (“Case Reports” [Publication Type] OR “Editorial” [Publication Type] OR “Review” [Publication Type]) AND “English” [Language] AND (2020:2024 [pdat])	1334

### Appendix A.2. Embase Search Strategy

Date Searched: 2 April 2025

**Set #**	**Concept**	**Syntax**	**Results**
1	AI	‘artificial intelligence’/exp OR ‘artificial neural network’/exp OR ‘image processing’/exp OR ‘deep learning’/exp OR ‘machine learning’/exp OR ‘Artificial Intelligence’:ti,ab,kw OR ‘Artificial Neural Network’:ti,ab,kw OR ‘Convolutional Neural Network’:ti,ab,kw OR ‘Deep Learning’:ti,ab,kw OR ‘Machine Learning’:ti,ab,kw	760,426
2	PET/MRI	1,696,145	921,043
3	Alzheimer	‘Alzheimer disease’/exp OR Alzheimer:ti,ab,kw OR Alzheimers:ti,ab,kw OR ‘Senile Dementia’:ti,ab,kw OR ‘Presenile Dementia’:ti,ab,kw	337,471
4	Diagnosis or detection	‘diagnosis’/exp OR diagnoses:ti,ab,kw OR diagnose:ti,ab,kw OR diagnosis:ti,ab,kw OR detect *:ti,ab,kw	13,097,987
5	combining	#1 AND #2 AND #3	5670
6	filters	#4 NOT (‘case report’/de OR [editorial]/lim OR [review]/lim) AND [2020–2025]/py AND [english]/lim AND [humans]/lim	2843

### Appendix A.3. Scopus Search Strategy

Date Searched: 2 April 2025

**Set #**	**Concept**	**Syntax**	**Results**
1	AI	TITLE-ABS-KEY (“Artificial Intelligence” OR “Artificial Neural Network” OR “Convolutional Neural Network” OR “Deep Learning” OR “Machine Learning”)	1,881,748
2	PET/MRI	TITLE-ABS-KEY (“Positron Emission Tomography” OR “PET” OR “Magnetic Resonance Imaging” OR MRI)	1,506,097
3	Alzheimer	TITLE-ABS-KEY (alzheimer OR alzheimers OR “Senile Dementia” OR “Presenile Dementia”)	316,171
4	Diagnosis or detection	TITLE-ABS-KEY (diagnoses OR diagnose OR diagnosis OR detect *)	13,530,301
5	combining	#1 AND #2 AND #3	3612
6	filters	#4 date limit 2020–2025 and English language	2848
